# Case report: Pilomatrix carcinoma with PDL1 expression and *CDKN2A* aberrant

**DOI:** 10.3389/fimmu.2024.1337400

**Published:** 2024-05-28

**Authors:** Ayinuer Abula, Sheng-Qiang Ma, Sisi Wang, Wei Peng, Xiaming Pei, Zhe-Yu Hu

**Affiliations:** ^1^ The Affiliated Cancer Hospital of Xiangya School of Medicine, Central South University/Hunan Cancer Hospital, Changsha, China; ^2^ Department of Oncology, Turpan City People’s Hospital, Tulufan, China; ^3^ Department of Oncology, The Second Xiangya Hospital, Central South University, Changsha, China; ^4^ Department of General Surgery, Turpan City People’s Hospital, Tulufan, China; ^5^ Department of Urinary Surgery, Turpan City People’s Hospital, Tulufan, China

**Keywords:** pilomatrix carcinoma, PDL1, PD1, CDKN2A, chemotherapy

## Abstract

**Case report:**

A 55-year-old male patient developed a mass in the left inguinal area with left lower limb swelling and first visited a local hospital 3 months earlier because of unrelieved pain. An MRI scan suggested left suprapubic branch and left acetabular bone destruction, abnormal soft tissue signals within the iliopsoas muscle of the anterior edge of the left iliac bone, and enlarged lymph nodes in the left iliac fossa and left inguinal region. The patient subsequently underwent left pelvic lesion open biopsy and inguinal lymph node resection biopsy. According to pathological reports, the left inguinal mass was considered to be a malignant tumor of cutaneous accessory origin (pilomatrix carcinoma) with extensive vitreous changes. The suprapupubis branch mass was considered to be a bone metastatic pilomatrix carcinoma. Immunohistochemistry (IHC) revealed a PDL1 combined positive score (CPS) of 8. DNA next-generation sequencing (NGS) showed *CDKN2A* L65Rfs*53 mutation. The patient received three cycles of gemcitabine and nedaplatin. However, the lesion progressed.

**Conclusion:**

Chemotherapy is not effective for treating pilomatrix carcinoma. PDL1 antibodies and CDK4/6 inhibitors might be treatment options for pilomatrix carcinoma.

## Introduction

Pilomatrix carcinoma (PC), a rare and locally aggressive malignancy, was first reported by Lopansri and Mihm in 1980 (1, 2). Pilomatrix carcinoma originates from hair follicle matrix cells and is a malignant variant of pilomatrixoma. To date, only 150 cases have been reported in the literature, and the most common site is the head and neck (3). Pilomatrix carcinoma frequently occurs in patients aged between 50 and 70 years (4–6). For 52 patients diagnosed in the last decade, the male-to-female ratio was 1.3:1 (7).

Local excision is recommended for local pilomatrix carcinoma (8). However, as the local recurrence rate after simple resection is relatively high, adjuvant radiotherapy is sometimes recommended. Although pilomatrix carcinoma is a low-grade malignancy and has a low metastatic propensity, lymph node metastasis and lung metastasis have been reported (9–11). Moreover, metastatic lesions are insensitive to chemotherapy, with poor prognosis (12).

Although several studies have described the epidemiologic features, clinical manifestations, traditional therapeutic options, and the prognosis of pilomatrix carcinoma, the molecular and biological characteristics of this disease have not yet been reported. In this study, we present the genetic abnormalities and immune characteristics of one patient with PC located in the left inguinal area with local lymph node and bone metastasis. The patient was informed that the data from his case would be submitted for publication, and he agreed.

## Case report

**Figure 1:**
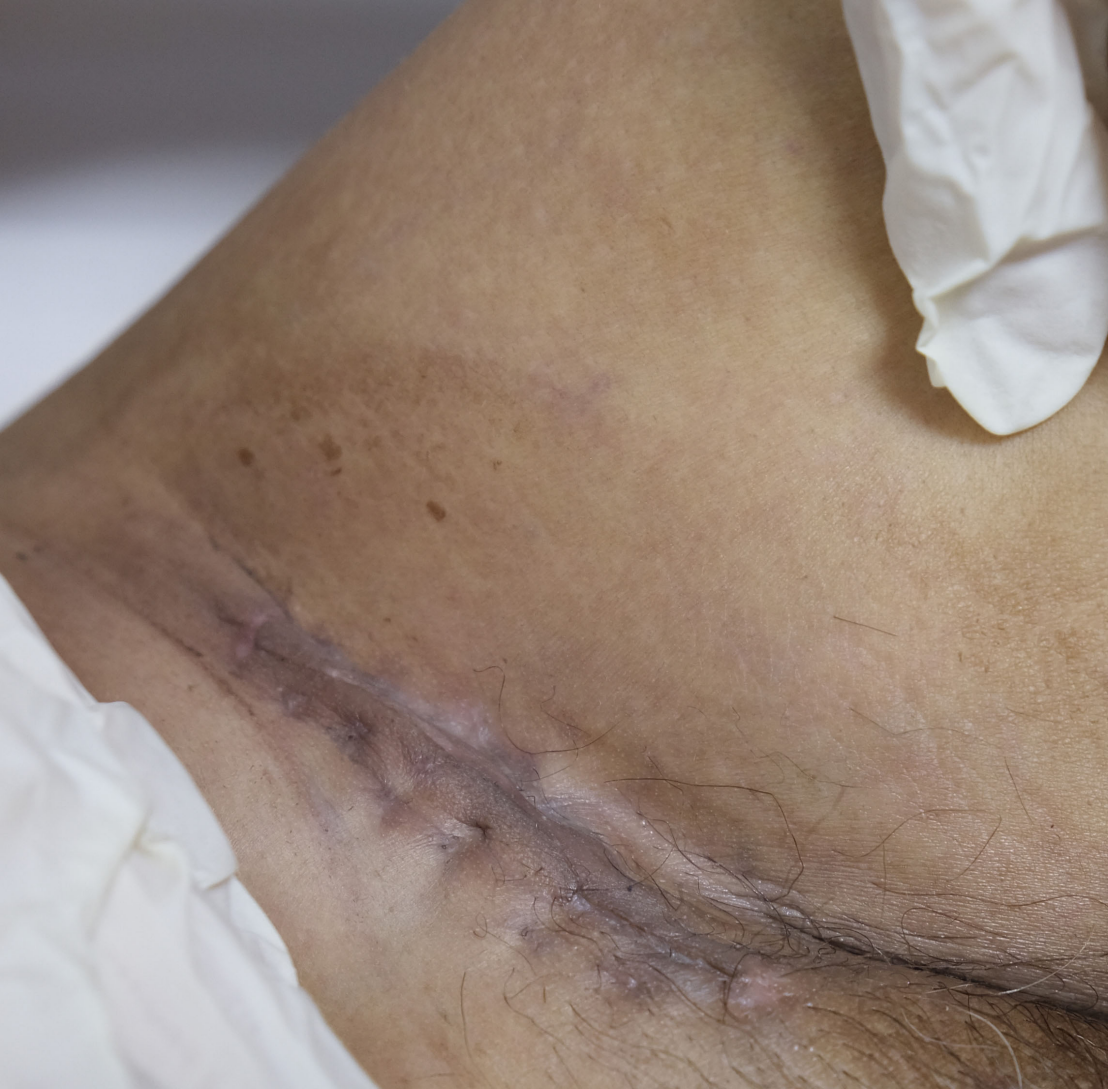
Photograph of the patient showing a soft, solitary, subcutaneous, and slow-growing mass (about 5 cm) in the left inguinal region with a sutured wound for biopsy surgery.

**Figure 2:**
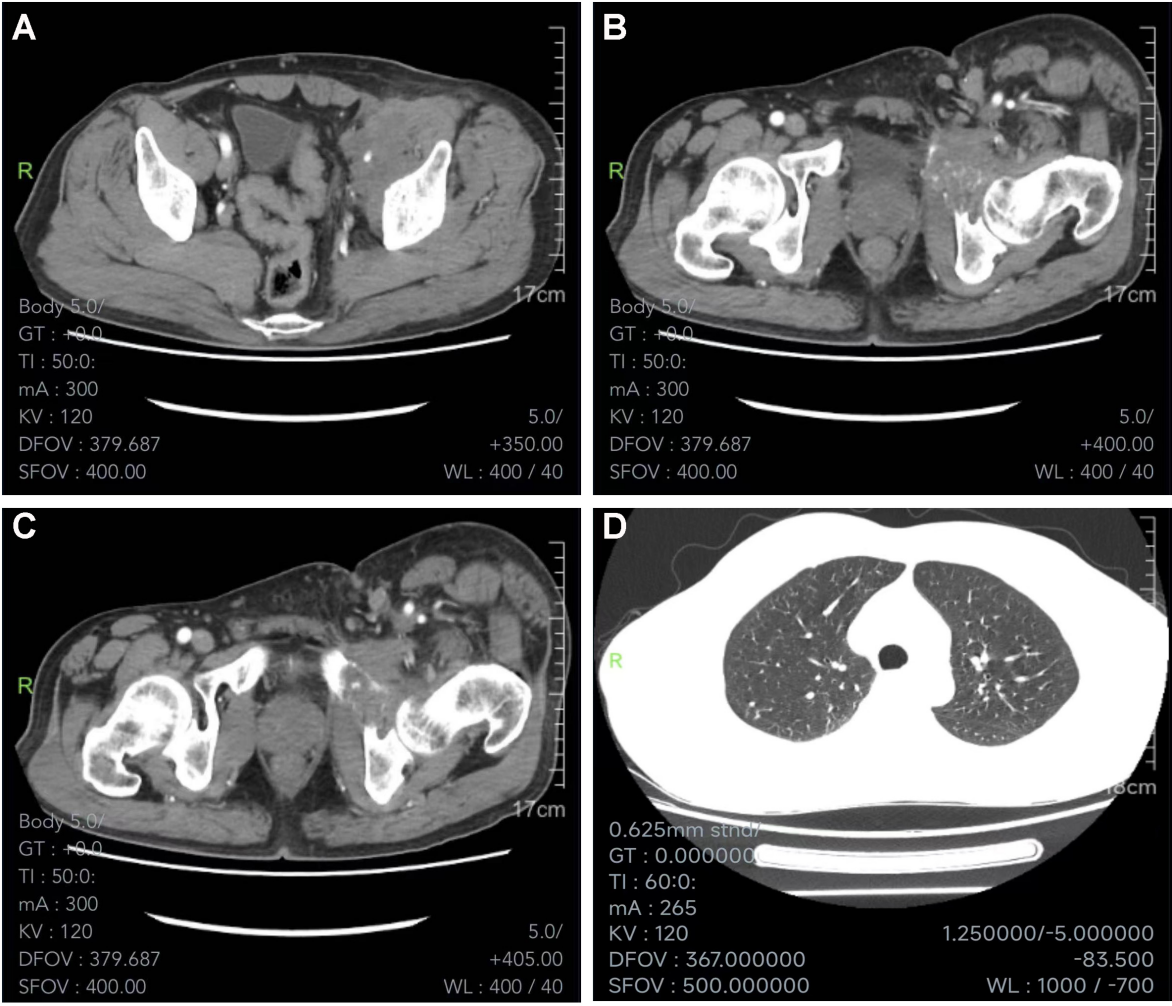
MRI and CT scan. **(A)** MRI scan of the abdominopelvic cavity showing irregular, slightly longer T1 and slightly longer T2 confounding signals in the iliosas muscle of the left iliac bone anterior edge. Its visible patchy short T2 signal, unclear boundary, and pressure lipid sequence was mixed with high signal, about 7.21 cm × 4.88 cm. The left iliac fossa and left inguinal area had scattered enlarged lymph nodes, with a larger diameter of about 1.6 cm. **(B, C)** MRI scan showing bone destructions in the left suprapuphysical branch and left acetabulum. **(D)** Chest CT showing multiple calcification foci and a few cables in the posterior segment of the upper lobe of the left lung and small nodules in the posterior segment of the right upper lung and the dorsal segment of the left lung. The larger diameter was about 3.4 mm; clear texture of the remaining lungs, no signs of stenosis and compression.

**Figure 3:**
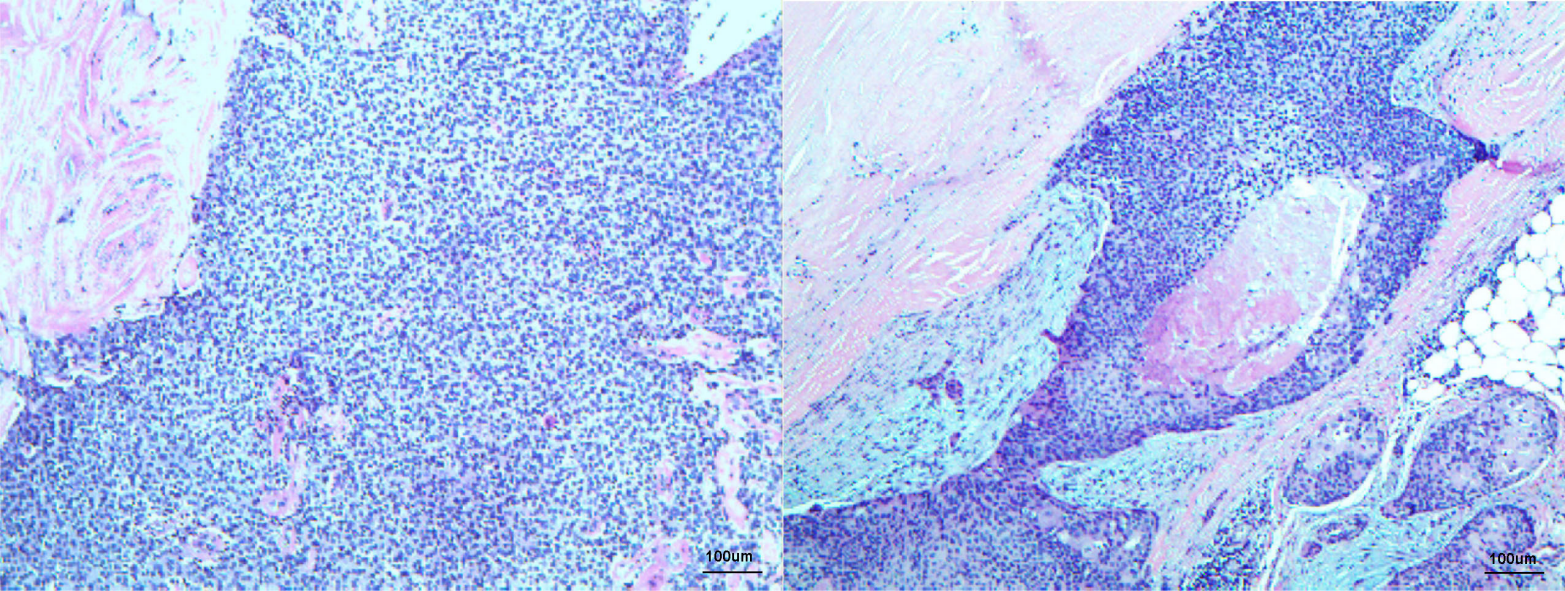
Histopathology displaying low-power microscopic view of left inguinal mass consisting of cutaneous accessory origin (atypical basaloid cells with frequent mitoses) with extensive vitreous changes fitting the diagnosis of pilomatrix carcinoma, H&E staining ×100 original magnification.

**Figure 4:**
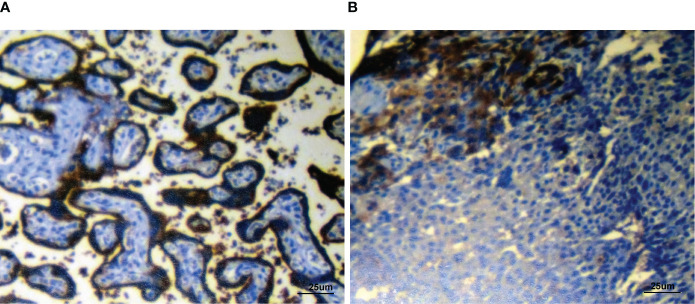
Immunohistochemical detection of PDL1 showing the positive control slide **(A)** and patient’s biopsy tissue **(B)** (PDL1 combined positive score (CPS): 8). Staining, ×400 original magnification.

A 55-year-old man had a soft, isolated, subcutaneous, slow-growing mass (approximately 5 cm) in the left inguinal region (**Figure 1**). When the mass was first detected 1 year earlier, he felt swelling without pain. The local hospital diagnosis was nerve compression, and no specific treatment was given. At 6 months earlier, the patient experienced pain in the left inguinal area. The pain worsened during activity and was relieved at rest, obviously affecting his daily life. A local hospital administered some conservative treatment, but the symptoms were not relieved. At 3 months earlier, the patient visited the Department of Bone Oncology, the First Affiliated Hospital of Xinjiang Medical University, and underwent imaging and pathology examinations. The MRI results showed the following: (**Figure 2**) the left suprapuphysical branch and left acetabulum had bone destruction; the iliosal muscle of the left iliac bone anterior edge had an irregular, slightly longer T1 and a slightly longer T2 confounding signal, a visible, patchy, short T2 signal, and an unclear boundary; the pressure lipid sequence was mixed with a high signal, approximately 7.21 cm × 4.88 cm; and the left iliac fossa and left inguinal area had scattered enlarged lymph nodes, with a larger diameter of approximately 1.6 cm. The chest CT showed multiple calcification foci and a few cables in the posterior segment of the upper lobe of the left lung and small nodules in the posterior segment of the right upper lung and the dorsal segment of the left lung; the larger diameter was approximately 3.4 mm, and there was a clear texture in the remaining lungs, with no signs of stenosis or compression (**Figure 2**). The patient subsequently underwent left pelvic lesion open biopsy and inguinal lymph node resection biopsy. The pathological examination revealed that the left inguinal mass was a malignant tumor of cutaneous accessory origin (pilomatrix carcinoma) with extensive vitreous changes. The suprapupubis branch mass was considered to be a bone metastatic pilomatrix carcinoma (**Figure 3**). Immunohistochemical detection revealed a PDL1 combined positive score (CPS) of 8 (**Figure 4**). High-throughput next-generation sequence (NGS) revealed a *CDKN2A* L65Rfs*53 mutation with a frequency of 33% in biopsy tissue, suggesting that abemaciclib or palbociclib might be effective for treating this tumor, but there is a lack of supporting evidence from clinical trials. Genetic testing revealed microsatellite stabilization (MSS) and a low tumor mutation burden (TMB-L, 1.0 muts/Mb).

A multidisciplinary team (MDT) suggested that radical resection was impossible due to the wide invasion regions and the highly destructive nature of the surgical procedure. Radiation also failed to affect the tumor region. Chemotherapy with gemcitabine plus platinum was recommended to narrow the scope of the tumor and provide the opportunity for later surgery. After three cycles of chemotherapy, the tumor had progressed.

## Discussion

Based on the most recent literature review of PC (13), only approximately 124 cases have been documented since the first report in 1980. Various studies have proposed a connection between pilomatrix carcinoma and anomalies within exon 3 of the β-catenin (*CTNNB1*) gene (12, 14–17), which encodes the β-catenin protein. β-Catenin functions as a 92-kDa adherens junction (AJ) protein, facilitating cell–cell adhesion through AJs. AJs convey a signal that indicates the presence of neighboring cells and stabilizes the actin cytoskeleton. Cells form and leave epithelia during multiple stages of embryogenesis, wound healing, and cancer metastasis. Disturbance and restoration of connections between epithelial cells are crucial for their function. Disassembly and assembly of AJs may regulate a process (18) in which β-catenin acts as an effector in the Wnt/β-catenin/Tcf-Lef pathway, promoting cellular differentiation and division. A.M. Hassanein et al. reported that 100% of PCs had strong nuclear and cytoplasmic β-catenin staining in the basaloid areas but that transitional areas only displayed cytoplasmic and membranous staining, with occasional nuclei showing some staining. The absence of staining was observed in shadow cells (17). Activation of the Wnt/Ctnnb1/Tcf-Lef pathway leads to the differentiation of normal matrix cells into hair shafts in hair follicles. *CTNNB1* mutations are present in both benign and malignant neoplasms across a diverse range. Alexander J.F. Lazar et al. reported that the mutations in exon 3 of the *CTNNB1* gene are concentrated near the codons that encode serine residues at positions 33 and 37 of the amino acid sequence (16). These mutations arise in the absence of any underlying issue with DNA mismatch repair (19). Involvement of β-catenin in the development of pilomatrix neoplasia suggests its direct contribution to tumorigenesis.

Other mutations in *ARID1A*, *PTEN*, and *PIK3CA* were detected in vulvar pilomatrix carcinoma (15), which might be responsible for the aggressive behavior of PC and related to poor outcomes (20). CDX2, LEF-1 and SATB2 are also positively expressed in pilomatrix carcinoma (21, 22). Several preliminary reports have demonstrated that in the presence of high PDL1 expression, systematic immunotherapy with an anti-PD1 antibody may result in a nearly complete response in patients with sebaceous carcinoma with brain metastases and metastatic prostate cancer (23–25). In the present case, PDL1 was positively expressed, suggesting that immunotherapy with the anti-PD1 agent pembrolizumab is a potential treatment option.

Cyclin D1 nuclear positivity in the basaloid cells of the tumors, apart from that of PDL1, varies from 10% to 50% (16). Cyclin D1 is the downstream target of the WNT/beta-catenin signaling pathway. Cyclin D1, when combined with cyclin-dependent kinases 4 and 6 (CDK4 and CDK6), plays a crucial role in controlling the progression of cellular division and acts as a major facilitator in protecting against cancer. Mutations in *CTNNB1*, *KRAS*, and *CDKN2A* are suggested to potentially serve as predictive indicators for the response to the CDK4/6 inhibitor abemaciclib (26). In the present case, the *CDKN2A* L65Rfs*53 mutation was detected in 33% of tumor cells, suggesting that a CDK4/6 inhibitor is another potential treatment option.

Surgical resection and radical radiation were not suitable for the patient in the present case. However, the tumor was not responsive to chemotherapy. PDL1 IHC and NGS results suggested chemotherapy plus an anti-PD1 antibody and a CDK4/6 inhibitor as potential treatment options.

*CDKN2A* L65Rfs*53 mutation might have some influence on the efficacy of the anti-tumor drug. However, so far, there are no reports of this mutation. We hope to have more reports on pilomatrix carcinoma so that the investigators can gather more evidence to study. Currently, there are no official guidelines for the treatment of pilomatrix carcinoma, and the indications for PD1/PDL1-antibodies also have not included this malignancy yet. Thus, the self-paid high price causes patients to refuse its use. Because pilomatrix carcinoma is a rare kind of malignancy, a large-scare clinical trial is unpractical. What we think is operational is that drug suppliers offer free drugs to these rare patients, observe the efficacy, and slowly move into the guidelines.

## Data availability statement

The original contributions presented in the study are included in the article/supplementary material. Further inquiries can be directed to the corresponding authors.

## Ethics statement

The studies involving humans were approved by the ethics committee of Turpan City People’s Hospital. The studies were conducted in accordance with the local legislation and institutional requirements. The human samples used in this study were acquired from examination results, pathological findings, and medical records. Written informed consent for participation was not required from the participants or the participants’ legal guardians/next of kin in accordance with the national legislation and institutional requirements. Written informed consent was obtained from the individual(s) for the publication of any potentially identifiable images or data included in this article.

## Author contributions

AA: Data curation, Writing – original draft, Funding acquisition. S-QM: Data curation, Validation, Visualization, Writing – original draft. SW: Data curation, Writing – original draft, Resources, Validation. WP: Data curation, Validation, Writing – original draft, Visualization. XP: Data curation, Validation, Visualization, Writing – original draft, Methodology. Z-YH: Data curation, Validation, Visualization, Writing – original draft, Conceptualization, Writing – review & editing.
